# Anti-Inflammatory Pyranochalcone Derivative Attenuates LPS-Induced Acute Kidney Injury via Inhibiting TLR4/NF-κB Pathway

**DOI:** 10.3390/molecules22101683

**Published:** 2017-10-10

**Authors:** Min Shi, Xiaoxi Zeng, Fan Guo, Rongshuang Huang, Yanhuan Feng, Liang Ma, Li Zhou, Ping Fu

**Affiliations:** Kidney Research Institute, Division of Nephrology, West China Hospital of Sichuan University, Chengdu 610041, Sichuan, China; minshi0616@163.com (M.S.); zxx5012@163.com (X.Z.); gf_9306@163.com (F.G.); huangrongshuang@126.com (R.H.); fengyanhuan89@163.com (Y.F.); zhouli126@hotmail.com (L.Z.)

**Keywords:** AKI, pyranochalcone, derivative, anti-inflammation, TLR4, NF-κB

## Abstract

Treatment of septic acute kidney injury (AKI) has still been beyond satisfaction, although anti-inflammatory therapy is beneficial for sepsis-induced AKI. Compound **5b** was derived from natural pyranochalcones and exhibited potent anti-inflammatory activity in adjuvant-induced arthritis. In this study, we aimed to investigate the renoprotective effects and potential mechanism of **5b** against lipopolysaccharide (LPS)-induced AKI. C57BL/6 mice and human renal proximal tubule cell line (HK-2 cell) were treated with LPS, respectively. Compound **5b** was orally administrated at a dose of 25 mg/kg/day for 5 days before LPS (10 mg/kg) intraperitoneal injection. Cells were pretreated with 25 μg/mL **5b** for 30 min before LPS (1 μg/mL) treatment. Pretreatment with **5b** markedly alleviated tubular injury and renal dysfunction in LPS-induced AKI. The expression of IL-1β, IL-6, and TNF-α both in renal tissue of AKI mice and in the LPS-stimulated HK-2 cell culture medium were reduced by **5b** treatment (*p* < 0.05). The results of immunohistochemistry staining showed that **5b** reduced the expression of NF-κB p65 in kidneys. Similarly, **5b** decreased the LPS-induced levels of NF-κB p65 and TLR4 proteins in kidneys and HK-2 cells. These data demonstrated that a potent pyranochalcone derivative, **5b**, exhibited renoprotective effect against LPS-induced AKI, which was associated with anti-inflammatory activity by inhibiting the TLR4/NF-κB pathway.

## 1. Introduction

Acute kidney injury (AKI), characterized by abrupt deterioration in renal function, is diagnosed by increased serum creatinine (SCr) or oliguria. AKI is associated with increased risk of cardiovascular disease, chronic kidney disease (CKD) and end stage renal disease (ESRD), and increased mortality [1,2]. It has been reported that AKI occurs in approximately half of patients with sepsis and increases mortality six- to eight-fold [3]. However, treatment of septic AKI has still been beyond satisfaction. Therefore, it is necessary to explore effective therapeutic options for sepsis-induced AKI.

Considerable studies have demonstrated that the inflammatory response plays a pivotal role in the pathogenesis of septic AKI [4,5]. Importantly, anti-inflammatory therapy was confirmed to be beneficial for the treatment of sepsis-induced AKI [6]. Endotoxin lipopolysaccharide (LPS) is a classical ligand for TLR4 and mediates TLR4-dependent signal transduction to activate NF-κB, leading to an increase in inflammatory cytokine expression such as interleukin-1β (IL-1β), interleukin-6 (IL-6), and tumor necrosis factor-α (TNF-α) [7,8]. Therefore, an LPS-induced experimental model was comprehensively used to investigate and evaluate the anti-inflammatory treatment of sepsis-related AKI.

**Figure 1:**
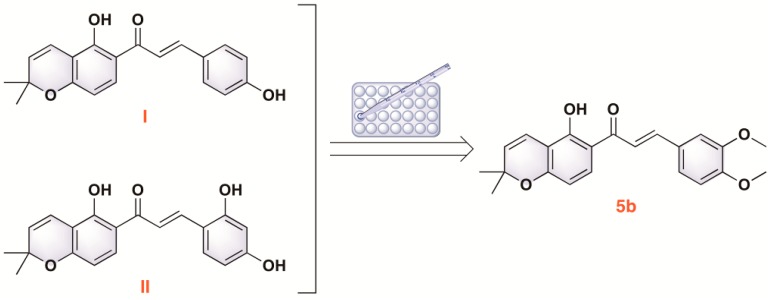
The chemical structure of **5b**.

Natural products from traditional herbal medicine have potential for the investigation of novel anti-inflammatory drugs [9,10]. In our previous study, two typical representatives of pyranochalcones, 4-hydroxyloncarpin (I) and 3″,3″-dimethylpyrano[3′,4′]2,4,2′-trihydroxychalcone (II), isolated from *Millettia ferruginea* and *Artocarpus communis*, were found to exhibit anti-inflammatory activity. It has been reported that pyranochalcones exhibit various pharmacological activities including antimicrobial activity, radical scavenging activity, and the inhibition of nicotinamide adenine dinucleotide (NADH) and matrix metalloproteinase-2 (MMP-2) secretion [11,12,13,14]. As part of our continuing effort to explore new chemical entities, a series of pyranochalcone derivatives were designed, synthesized, and evaluated [15]. Among these derivatives, compound **5b** significantly suppressed acute inflammatory responses in adjuvant-induced arthritis (Figure 1). However, the renoprotective effects of **5b** against LPS-induced AKI remain unclear. In the study, we aimed to investigate the renoprotective function and potential mechanism of pyranochalcone derivative **5b** in LPS-induced AKI in vivo and in vitro.

## 2. Results

### 2.1. Effect of ***5b*** on LPS-Induced AKI Mice

**Figure 2:**
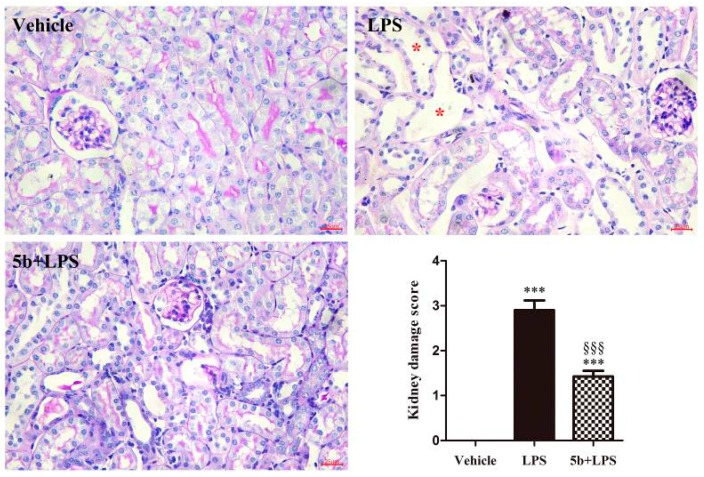
Effect of **5b** on lipopolysaccharide (LPS)-induced acute kidney injury (AKI) mice. Periodicacid-Schiff (PAS)-stained sections showed no damage in the vehicle group, the tubular injury in LPS group, and attenuated tubular injury in the **5b** + LPS group. Note there were many dilated tubules (asterisk) in LPS-induced AKI mice. Kidney damage score was expressed as means ± SD for groups of six mice. *** *p* < 0.001 vs. vehicle group; ^§§§^ *p* < 0.001 vs. LPS group.

Periodicacid-Schiff (PAS) staining showed normal kidney tubules in the vehicle group. In contrast, LPS-induced renal histopathological changes, such as swollen renal tubular epithelial cells, dilation of renal tubules, destructed tubular structures, and renal interstitial edema of epithelial cells, were observed (Figure 2). Importantly, pretreatment with **5b** (25 mg/kg/day for 5 days) alleviated tubular injury.

### 2.2. Effect of ***5b*** on Renal Function in Mice

**Figure 3:**
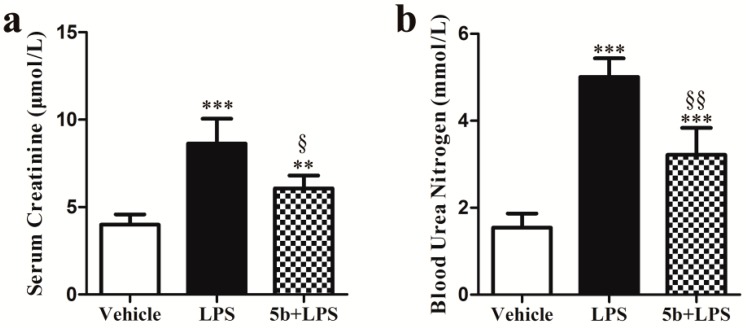
Effect of **5b** on SCr (**a**) and blood urea nitrogen (BUN) (**b**) in mice. Data were expressed as means ± SD for groups of six mice. ** *p* < 0.01 and *** *p* < 0.001 vs. vehicle group; ^§^ *p* < 0.05 and ^§§^ *p* < 0.01 vs. LPS group.

Levels of SCr and blood urea nitrogen (BUN) were significantly increased in LPS-injected mice in comparison with those of vehicle mice (SCr: *p* < 0.001; BUN: *p* < 0.001) (Figure 3). Pretreatment with **5b** significantly decreased levels of SCr and BUN in mice (SCr: *p* < 0.05 vs. LPS group; BUN: *p* < 0.01 vs. LPS group).

### 2.3. Effect of ***5b*** on Proinflammatory Cytokine Gene Expressions in Renal Tissue

**Figure 4:**
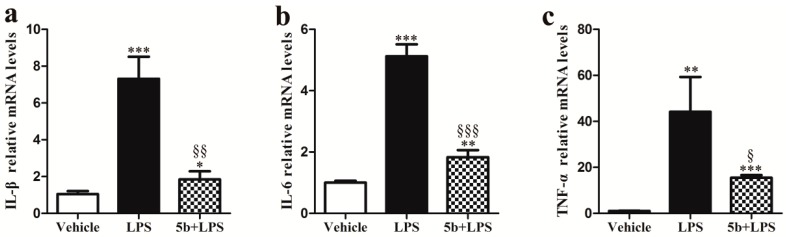
Effect of **5b** on gene expression levels of IL-1β (**a**), IL-6 (**b**), and TNF-α (**c**) in renal tissue. Data were expressed as means ± SD for groups of six mice. * *p* < 0.05, ** *p* < 0.01, and *** *p* < 0.001 vs. vehicle group; ^§^ *p* < 0.05, ^§§^ *p* < 0.01, and ^§§§^ *p* < 0.001 vs. LPS group.

The real-time PCR data exhibited that the gene expression levels of IL-1β, IL-6, and TNF-α in renal tissue significantly increased with the injection of LPS (Figure 4), while the pretreatment of **5b** reduced LPS-induced proinflammatory cytokine gene expressions in mice (*p* < 0.05). The results indicated that compound **5b** could inhibit excessive renal proinflammatory cytokine production in LPS-induced AKI mice.

### 2.4. Effect of ***5b*** on TLR4/NF-κB Pathway in LPS-Induced AKI Mice 

**Figure 5:**
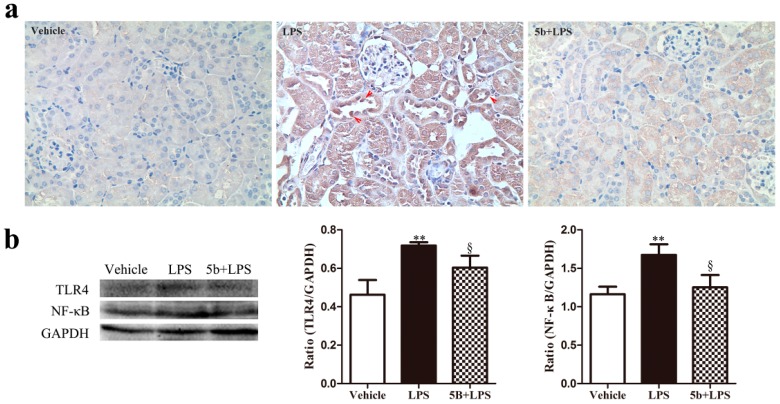
Effect of **5b** on the expression of NF-κB p65 and TLR4 in the kidneys of mice. Expression of NF-κB p65 was measured using immunohistochemistry staining. (**a**) Levels of NF-κB p65 and TLR4 proteins were measured using Western blot analysis; (**b**) Data were expressed as means ± SD for groups of six mice. ** *p* < 0.01 vs. vehicle group; ^§^ *p* < 0.05 vs. LPS group.

As the TLR4/NF-κB pathway is closely related to the abnormal release of proinflammatory cytokines, we further explored the regulation of **5b** on the activation of the TLR4/NF-κB pathway in renal tissue. Immunohistochemistry staining exhibited that the renal expression of NF-κB p65 in nuclei and cytoplasm of tubular cells was greater in the LPS group than that in the vehicle group (Figure 5). Importantly, oral administration of **5b** reduced the renal expression of NF-κB p65 in LPS-induced AKI. Additionally, the protein expression of NF-κB p65 and TLR4 in renal tissues increased by LPS treatment, while pretreatment with **5b** for 5 days effectively downregulated the levels of NF-κB p65 and TLR4 proteins. These results highlighted that compound **5b** exerted a renoprotective effect on LPS-induced AKI mice through the inhibition of the TLR4/NF-κB pathway.

### 2.5. Effect of ***5b*** on Proinflammatory Cytokines in LPS-Induced HK-2 Cells

**Figure 6:**
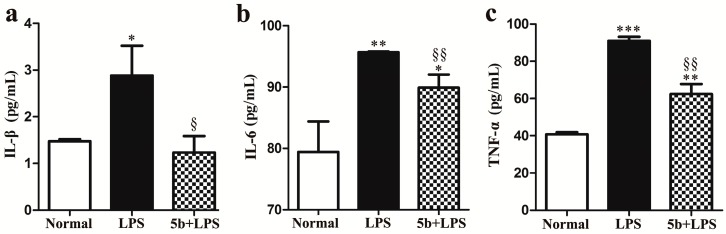
Effect of **5b** on IL-1β (**a**); IL-6 (**b**); and TNF-α (**c**) release induced by LPS in HK-2 cells. HK-2 cells were treated with LPS with or without **5b**. Then, 100 μl of culture medium in each sample (groups of three samples) was taken out to measure the levels of IL-1β, IL-6, and TNF-α using ELISA kits. Data were expressed as means ± SD for three independent experiments. * *p* < 0.05, ** *p* < 0.01 and *** *p* < 0.001 vs. normal group; ^§^ *p* < 0.05 and ^§§^ *p* < 0.01 vs. LPS group.

The levels of IL-1β, IL-6, and TNF-α were determined by ELISA after 24 h of treatment with LPS with or without **5b**. As shown in Figure 6, LPS-induced the excretion of proinflammatory cytokines IL-1β, IL-6, and TNF-α in HK-2 cells, while compound **5b** suppressed the release of IL-1β, IL-6, and TNF-α in LPS-stimulated HK-2 cells.

### 2.6. Effect of ***5b*** on TLR4/NF-κB Pathway in LPS-Induced HK-2 Cells 

**Figure 7:**
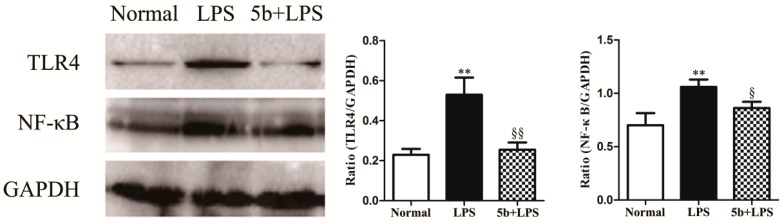
Effect of **5b** on expression of NF-κB p65 and TLR4 in HK-2 cells. Expression of NF-κB p65 and TLR4 proteins were assessed by Western blot analysis. Data were expressed as means ± SD for three independent experiments. ** *p* < 0.01 vs. normal group; ^§^ *p* < 0.05 and ^§§^ *p* < 0.01 vs. LPS group.

To investigate the role of the TLR4/NF-κB pathway in **5b**-treated HK-2 cells, the expression levels of NF-κB p65 and TLR4 proteins were detected by Western blotting. As exhibited in Figure 7, treatment of LPS upregulated NF-κB p65 and TLR4 proteins in HK-2 cells, while **5b** significantly decreased the expression of the corresponding proteins. These results also suggested that **5b** possessed a protective effect on LPS-stimulated HK-2 cells via inhibiting TLR4/NF-κB.

## 3. Discussion

AKI is a serious and frequent complication in patients with sepsis in intensive care units (ICUs) [4,16]. It has been demonstrated that sepsis and septic shock are the leading causes of AKI in ICUs and that they associated with a high mortality [4,17]. The pathophysiology of septic AKI is complex, in which inflammation response plays a key role [4,5,18]. Despite extensive investigations, there is no effective therapeutic strategy against sepsis-induced AKI. In the study, we investigated the effects of a natural product, pyranochalcone-derived **5b**, on LPS-induced AKI in mice and in HK-2 cells.

We established a septic AKI experimental model by treating male C57BL/6 mice with intraperitoneal injection of 10 mg/kg LPS, according to previous studies [8,19]. The kidney damage score of LPS-treated mice was 2.90 ± 0.22, and the levels of SCr and BUN exhibited respective two-fold and three-fold increases, compared to vehicle-treated mice. Pretreatment with **5b** at a dose of 25 mg/kg/day for 5 days significantly improved histological damage and renal dysfunction in LPS-induced AKI, indicated by reduced kidney damage score (1.50 ± 0.13), as well as SCr and BUN levels, respectively. These results confirmed that a suitable LPS-induced AKI model was successfully developed and **5b** exhibited potent renoprotective effect against LPS-induced AKI.

Numerous studies have demonstrated that proinflammatory cytokines have prominent roles in sepsis-induced AKI [19,20]. Excessive release of inflammatory factors, such as IL-1β, IL-6, and TNF-α, triggered pathophysiological abnormities of sepsis [19,21,22,23]. Therefore, anti-inflammatory therapy could be effective for the treatment of septic AKI, and novel chemical entities or drug candidates with anti-inflammatory activity would be beneficial for sepsis-induced AKI [6,8,19,24,25]. In our previous study, we designed and synthesized natural product, pyranochalcone-derived analogs, which were further evaluated by anti-inflammatory assay. In adjuvant-induced arthritis and carrageenan-induced hind paw edema experimental models, compound **5b** effectively inhibited the progression of inflammation [15]. Our results showed that pretreatment with **5b** decreased the renal expression of proinflammatory cytokines IL-1β, IL-6, and TNF-α in LPS-induced AKI, which suggested that the protection of **5b** against LPS-induced AKI was associated with the downregulation of IL-1β, IL-6, and TNF-α levels. Renal tubular epithelial cells are a critical factor in renal injury because they exhibit immune response characteristics during the sepsis-induced AKI process [26,27]. In LPS-induced AKI, inflammatory cytokines are upregulated in renal tubular epithelial cells and then participate in AKI inflammatory responses [28,29,30]. In our study, **5b** treatment reduced the release of these inflammatory cytokines induced by LPS in HK-2 cells, which was consistent with the result in vivo study.

Toll-like receptor 4 is a pattern recognition receptor that functions as an LPS sensor, and whose activation recruits inflammatory factors and leads to kidney injury [31,32]. Furthermore, the activation of TLR4 sensitizes the NF-κB pathway, related to initiating the expression of proinflammatory cytokines IL-1β, IL-6, and TNF-α [8,33,34]. The activation of the TLR4/NF-κB pathway has been identified as a major contributor of inflammation in the kidney [8]. Moreover, the inhibition of the TLR4/NF-κB-mediated inflammatory response has been confirmed to have renoprotective effects against LPS-induced AKI [8,25,32]. Possible mechanisms by which some products inhibit the TLR4/NF-κB pathway include targeting the inhibitor of nuclear factor kappa-B kinase (IKK) and inhibiting other enzymes [35]. In this study, our results highlighted that treatment with **5b** inhibited the expression of NF-κB p65 and TLR4 proteins in vivo and in vitro. This indicated that **5b** suppressed the TLR4/NF-κB signaling pathway to reduce LPS-induced AKI.

## 4. Materials and Methods

### 4.1. Animal and Treatment

Male C57BL/6 mice (8–10 weeks old; 25–30 g) were purchased from the Animal Laboratory Center of Sichuan University (Chengdu, China). Mice had free access to water and food. Mice (*N* = 18) were randomly divided into three groups of six mice each. LPS-induced AKI animal models were generated by the intraperitoneal injection (i.p.) of LPS (Sigma Aldrich, Shanghai, China) at a dosage of 10 mg/kg in 200 μL saline for 18 h. To investigate the protective effect of **5b** on LPS-induced AKI, **5b** dissolved in 30% PEG400 was orally administered to mice at a dose of 25 mg/kg/day for 5 days before i.p. injection of LPS. Control animals received the same volume of vehicle (vehicle group). Groups of six mice were sacrificed 18 h after treatment with LPS. Blood samples and kidney tissues were collected for further investigations. Animal experimental protocols were approved by Animal Care and Use Ethics Committee of Committee of Sichuan University in China (IACUC number: 20100318).

### 4.2. Measurement of Renal Function

Blood samples were obtained from the heart under anesthesia. Levels of serum creatinine (SCr) and blood urea nitrogen (BUN) were measured to evaluate renal function. Level of SCr was measured by the method of HPLC-MS/MS. BUN was determined by a Chemistry Analyzer.

### 4.3. Histological Examination

Kidney tissues were fixed in 10% neutral buffered formalin, embedded in paraffin, then sectioned (4 μm) and stained with periodicacid-Schiff (PAS). Renal tubular injury was evaluated using the previously described 0–4 semiquantitative scale [36]. Briefly, ten fields (×400) of cortical tissues per animal were counted, and the percentage of death area in which epithelial necrosis, tubular dilation, and loss of brush boarder were observed was determined, in which 0, normal kidney; 1, <25%; 2, 25–50%; 3, 50–75%; 4, >75%. 

### 4.4. Immunohistochemistry

Heat-induced epitope retrieval was performed on dewaxed slides in citrate buffer (pH 6.0) at 95 °C for 40 min. Then, sections were exposed to peroxidase blocking solution (3% H_2_O_2_) prior to the addition of the primary antibody, anti-NF-κB p65 antibody (Abcam, Cambridge, MA, USA) diluted to 1:200 in PBS. After incubation with the primary antibody overnight at 4 °C, the slides were washed three times with PBS, and incubated with horseradish peroxidase (HRP)-incubated secondary antibody (Abcam, Cambridge, MA, USA) for 45 min. The sections were washed again with PBS three times. Subsequently, the slides were developed by diaminobenzidine (DAB) and counterstained with hematoxylin. Finally, the slides were observed under a light microscope.

### 4.5. Quantitative Real-Time PCR Analysis

Total RNA from renal tissues was isolated using a total RNA extraction Kit (Biotek, Winooski, VT, USA) according to the experimental protocols. Then, the concentration of mRNA was measured using a ScanDrop 100 (Analytik Jena, Thuringia, Germany) determiner. After reverse transcription, quantitative real-time PCR was performed using the fast qPCR kit (Kapa Biosystems, Foster, CA, USA) in a PCR system (CFX Connect; Bio-Rad, Hercules, CA, USA). Primer sequences are listed in Table 1. Relative gene expression was normalized with GAPDH and calculated using the 2^−ΔΔCt^ method.

### 4.6. Cell Culture

Human renal proximal tubule cell line (HK-2 cell) was a gift from Prof. Xueqing, Yu (The First Affiliated Hospital, Sun Yat-sen University). Cells were cultured in phenol red-free Dulbecco’s modified Eagle’s medium DMEM/F12 (Hyclone, Beijing, China) supplemented with 10% fetal bovine serum (FBS) in a humidified atmosphere of 5% CO_2_ at 37 °C. Cells were pretreated with 25 μg/mL **5b** for 30 min before treatment of LPS (1 μg/mL). After 24 h, the medium was collected to quantify the concentration of IL-1β, IL-6, and TNF-α.

### 4.7. Enzyme-Linked Immunosorbent Assay (ELISA)

The concentration of IL-1β, IL-6, and TNF-α in the culture medium was determined using human ELISA kits (Neobioscience, Shenzhen, China) following the manufacturer’s instructions.

### 4.8. Western Blot Analysis

Proteins were isolated from renal tissues or HK-2 cells with a RIPA lysis buffer and then analyzed by Western blotting. In brief, equal amounts of protein were separated by SDS-PAGE and then transferred on to a PVDF membrane (Bio-Rad, Hercules, CA, USA). Subsequently, the membranes were incubated with primary antibodies against NF-κB p65 (Abcam, Cambridge, MA, USA), TLR4 (Abcam, Cambridge, MA, USA), and GAPDH (Origene, Rockville, MD, USA) overnight at 4 °C. Then the membranes were incubated with HRP-conjugated secondary antibodies (R&D Systems, Minneapolis, MN, USA) for 1 h at room temperature. Finally, the proteins on the membrane were developed with an enhanced chemiluminescence agent (Millipore Corporation, Boston, MA, USA). The signals were detected using an Odyssey Infrared Imaging System (Bio-Rad, ChemiDoc MP, mANUSC, Bio-Rad Laboratories Inc., Hercules, CA, USA) and analyzed with the Image J program (National Institutes of Health, Bethesda, MD, USA). The ratio for the protein was normalized against GAPDH. 

### 4.9. Statistical Analysis

Data were expressed as means ± standard deviation (SD) and results were analyzed using SPSS 16.0 software (SPSS Inc., Chicago, IL, USA). One-way analysis of variance (ANOVA) followed by the Student-Newman-Keuls (SNK) test method was used to make comparison. For categorical variables, Kruskal-Wallis followed by Dunn’s post hoc analysis method was used. The level *p* < 0.05 was considered significant. 

## 5. Conclusions

In conclusion, pyranochalcone-derived **5b** exhibited potent renoprotective effect against LPS-induced AKI. Moreover, the renoprotective effect was associated with its anti-inflammatory function by inhibiting the TLR4/NF-κB pathway.

## Figures and Tables

**Table 1:** Sequences of the primers for real-time PCR.

Mouse Gene	Sequence (5′-3′)
*F-IL-1β*	TGGGCCTCAAAGGAAAGAAT
*R-IL-1β*	CAGGCTTGTGCTCTGCTTGT
*F-IL-6*	ACAACCACGGCCTTCCCTACTT
*R-IL-6*	CACGATTTCCCAGAGAACATGTG
*F-TNF-α*	ACCCTCACACTCAGATCATCTTC
*R-TNF-α*	TGGTGGTTTGCTACGACGT
*F-GAPDH*	GTATGACTCCACTCACGGCAAA
*R-GAPDH*	GGTCTCGCTCCTGGAAGATG
